# Advancing osteoarthritis research: the role of AI in clinical, imaging and omics fields

**DOI:** 10.1038/s41413-025-00423-2

**Published:** 2025-04-22

**Authors:** Jingfeng Ou, Jin Zhang, Momen Alswadeh, Zhenglin Zhu, Jijun Tang, Hongxun Sang, Ke Lu

**Affiliations:** 1 Shenzhen Hospital, Southern Medical University, Shenzhen, China; 2 Faculty of Computer Science and Control Engineering, Shenzhen University of Advanced Technology, Shenzhen, China; 3 Department of Orthopaedic Surgery, The First Affiliated Hospital of Chongqing Medical University, Chongqing, China

**Keywords:** Diseases, Bone

## Abstract

Osteoarthritis (OA) is a degenerative joint disease with significant clinical and societal impact. Traditional diagnostic methods, including subjective clinical assessments and imaging techniques such as X-rays and MRIs, are often limited in their ability to detect early-stage OA or capture subtle joint changes. These limitations result in delayed diagnoses and inconsistent outcomes. Additionally, the analysis of omics data is challenged by the complexity and high dimensionality of biological datasets, making it difficult to identify key molecular mechanisms and biomarkers. Recent advancements in artificial intelligence (AI) offer transformative potential to address these challenges. This review systematically explores the integration of AI into OA research, focusing on applications such as AI-driven early screening and risk prediction from electronic health records (EHR), automated grading and morphological analysis of imaging data, and biomarker discovery through multi-omics integration. By consolidating progress across clinical, imaging, and omics domains, this review provides a comprehensive perspective on how AI is reshaping OA research. The findings have the potential to drive innovations in personalized medicine and targeted interventions, addressing longstanding challenges in OA diagnosis and management.

## Introduction

OA is a chronic degenerative joint disease, primarily affecting weight-bearing joints such as the knees and hips. With the increasing prevalence of aging populations and rising obesity rates, the global burden of OA has surged, currently affecting over 500 million people.^1^ OA is characterized by complex pathological processes involving the degeneration of cartilage, bone remodeling, formation of osteophytes, and joint inflammation, ultimately leading to impaired joint function.^2^ It is a major contributor to chronic pain and disability, significantly diminishing quality of life and imposing considerable economic costs on both individuals and society.^3–5^

OA is primarily diagnosed through clinical symptoms, physical examination, and imaging, with radiography being the most common diagnostic tool.^6^ While X-rays are useful for detecting advanced structural damage such as joint space narrowing, osteophytes, and subchondral bone sclerosis, these findings often indicate late-stage disease.^7^ Early OA, characterized by molecular and biochemical changes in cartilage and synovial tissues, often remains undetected with conventional imaging methods.^8^ Although magnetic resonance imaging (MRI) can provide more detailed visualization of soft tissues, including cartilage, its high cost and limited accessibility reduce its routine use in clinical practice.^9^ Additionally, the interpretation of radiographic images is subject to interobserver variability, contributing to inconsistencies in diagnosis and disease grading.^10^ The widely adopted Kellgren-Lawrence grading scale, though useful for classifying OA severity based on radiographic findings, fails to detect early joint changes and lacks sensitivity to subtle, progressive deterioration.^11^

Therapeutically, current OA treatments are mainly symptomatic, focusing on pain management and inflammation reduction rather than addressing the underlying disease mechanisms.^12^ Pharmacological treatments, such as nonsteroidal anti-inflammatory drugs and intra-articular corticosteroid injections, provide short-term relief but do not modify the disease’s progression.^13^ More invasive options, including joint replacement surgery, are reserved for end-stage disease when the damage is irreversible, underscoring the need for earlier diagnosis and more effective therapeutic strategies.^14^ These limitations highlight the importance of developing more precise and predictive diagnostic tools and personalized treatments to address the multifactorial nature of OA.^15^

Recent developments in AI have shown potential in overcoming several of the limitations inherent in traditional OA diagnosis and treatment. AI algorithms, particularly deep learning models such as convolutional neural networks (CNNs), have demonstrated their ability to analyze large-scale medical images, identifying subtle structural changes in joint tissues that are often undetectable using conventional radiography.^16^ AI models can not only detect early-stage OA by analyzing cartilage thinning, bone abnormalities, and other microstructural alterations but also offer consistent, objective and reproducible assessments, reducing interobserver variability.^17^ For example, machine learning-based models have been developed to automatically grade OA severity from radiographic images with high accuracy, outperforming traditional methods.^18^ Spatial analysis of the OA microenvironment, using advanced techniques such as imaging and multi-omics approaches, has revealed critical information about the interactions between different tissue types, cells and signaling pathways that contribute to disease progression.^19–21^ In this context, the engineering of bone/cartilage organoids has emerged as a novel platform for recapitulating the cellular and molecular environments of OA. These organoids, which combine cells from both bone and cartilage, offer a promising tool for studying disease mechanisms in a controlled laboratory setting, as well as for testing potential interventions.^22^

AI is also revolutionizing OA progression prediction by integrating diverse datasets, including clinical, genetic, and biomechanical data.^23^ These models can identify high-risk patients by analyzing patterns that may indicate faster disease progression, allowing for earlier and more personalized interventions. Furthermore, AI is playing a crucial role in drug discovery, where machine learning techniques are used to predict novel therapeutic targets and repurpose existing drugs for OA treatment.^24^ Additionally, AI has shown unique applications in rehabilitation and regenerative medicine. AI-assisted rehabilitation technologies, such as those utilized in Winter Paralympics sports robots, enhance recovery by optimizing motor function training for patients with OA. Moreover, AI combined with advanced 3D bioprinting technologies optimizes biomaterial parameters for cartilage regeneration, paving the way for personalized treatments in regenerative OA therapy. These advances indicate that AI has the potential to significantly enhance OA diagnosis, treatment, and management, ultimately leading to more precise and personalized care for patients.

## Traditional diagnostic and research approaches in osteoarthritis

OA research and clinical practice have traditionally relied on a combination of clinical symptoms, imaging techniques, and biological data to assess disease progression, diagnose early signs of joint degeneration, and predict long-term outcomes.^25^ While these approaches provide valuable insights into the disease, they also have significant limitations, particularly in their ability to detect early-stage OA and quantify the disease’s complex progression.^26^ Below, we outline the key methods used in OA research, along with their roles in early detection, diagnosis, and prognosis, as well as their inherent limitations.

### Clinical cases and basic physiological information

#### Data requirements

Clinical symptoms, such as pain intensity, joint stiffness, and functional limitations, are essential metrics for evaluating the severity of OA and monitoring treatment efficacy. Pain is a core symptom of OA and is often the primary reason patients seek medical attention.^27^ Detailed information about the location, frequency, and intensity of pain, combined with assessments of stiffness and mobility restrictions, provides critical data for diagnosing OA and assessing its progression. Functional limitations, such as difficulty with activities like walking or bending, are equally important for understanding the disease’s impact on quality of life.^28^ Collecting these data helps clinicians evaluate the overall burden of disease and informs decisions on treatment strategies. In early screening, subtle increases in pain or decreases in mobility can signal the onset of OA, enabling earlier intervention.

#### Traditional applications

Traditionally, clinical data are collected through patient self-reports, often using standardized questionnaires such as the Western Ontario and McMaster Universities Osteoarthritis Index (WOMAC) or the Visual Analog Scale.^29^ Physical examinations complement patient feedback by evaluating joint tenderness, range of motion, and crepitus. These assessments help gauge disease severity and guide treatment decisions, including whether to recommend conservative management, pharmacological interventions, or surgery. For prognosis, consistent monitoring of clinical symptoms provides valuable insights into disease progression, facilitating adjustments to therapeutic approaches over time.

#### Limitations of traditional methods

Despite their utility, traditional methods for evaluating clinical symptoms are highly subjective and prone to bias. Patient self-reports can vary significantly depending on factors like pain tolerance, mood, and cultural differences, which complicates efforts to quantify the true impact of the disease.^30^ Physical examinations rely on the clinician’s experience and skill, introducing variability in diagnosis and staging. This issue is especially problematic in early osteoarthritis (OA), where symptoms may be mild or intermittent. Furthermore, traditional methods have difficulty capturing small, progressive changes in the disease’s course, making it challenging to monitor subtle OA progression over time.

### Imaging evaluation

#### Imaging modality

Imaging techniques are integral to diagnosing, detecting early signs, and monitoring OA, offering detailed insights into joint structures and surrounding tissue changes. The four most commonly utilized imaging modalities—X-ray (radiography), MRI, computed tomography (CT), and ultrasound (US)—each have distinct advantages and limitations:

- **X-ray:**
  A widely accessible and cost-effective imaging method, X-rays are primarily used to assess bone alterations, such as osteophyte formation, joint space narrowing, and bone remodeling, which are key indicators of OA progression. X-rays provide high-resolution images of bone structures, making them invaluable for detecting abnormalities like joint space reduction and osteophyte presence.^31^
- **MRI:**
  Known for its high sensitivity and specificity, MRI provides comprehensive visualization of soft tissue structures, including cartilage, ligaments, tendons, and the synovium. It accurately depicts joint damage and is particularly effective for identifying early OA signs such as cartilage thinning and subtle bone marrow lesions, often undetectable on X-rays.^32^
- **CT:**
  CT offers high-resolution bone imaging and excels at identifying osseous changes related to OA, including osteophyte formation and subchondral bone sclerosis. Its three-dimensional imaging capability is particularly valuable for evaluating bone density changes and the precise geometry of small bony structures, critical for assessing subchondral bone integrity and quantifying bone mineral density.^33,34^
- **US:**
  US provides real-time imaging of soft tissues, making it ideal for assessing synovitis, joint effusions, and the integrity of tendons and ligaments surrounding the joint. Its portability and real-time imaging capabilities enhance its utility for diagnostic and therapeutic interventions, such as guiding intra-articular injections.^35,36^

#### Data analysis

In traditional research, imaging data are employed to track and quantify OA progression. X-ray analysis often involves measuring joint space width to determine the degree of cartilage loss, a common method for assessing OA severity.^37^ The K-L grading system is widely used for this purpose, classifying OA severity from grades 0 to 4 based on radiographic features such as joint space narrowing, osteophyte formation, and subchondral sclerosis.^38^ MRI data analysis includes quantifying cartilage volume and assessing tissue composition to detect early degenerative changes.^39^ Scoring systems like the Whole-Organ Magnetic Resonance Imaging Score and the MRI Osteoarthritis Knee Score are employed to evaluate cartilage degradation, synovitis, bone marrow lesions, and other joint pathologies.^40,41^ CT scans provide detailed bone structure analysis, which is essential for assessing changes in bone density and the detailed geometry of osteophytes and subchondral bone regions commonly affected by OA. Bone texture metrics derived from CT images can predict areas vulnerable to further degenerative changes.^42^ US is used to assess joint effusions, synovitis, and soft tissue integrity around the joint. Semi-quantitative US scoring systems evaluate the severity of these features, which are indicative of inflammation and other pathological changes.^43,44^ US imaging is frequently analyzed to assess soft tissue alterations and fluid accumulation, offering immediate diagnostic insights.^45–47^

#### Limitations of traditional methods

The interpretation of these images is highly subjective and heavily relies on the clinician’s expertize, leading to potential variability in diagnosis among different observers.^48^ Furthermore, traditional methods often struggle to detect early tissue changes due to their limited sensitivity and resolution. For instance, subtle changes in cartilage composition might not be apparent until significant tissue damage has occurred. Additionally, traditional analysis methods might not effectively capture the complex, nonlinear relationships between different types of imaging data, potentially overlooking crucial diagnostic information.^49^

### Osteoarthrotomic data

#### Sequencing technology

The advent of advanced sequencing technologies has profoundly impacted OA research, allowing for both bulk and single-cell data collection to unravel the complexities of the disease at the molecular level. These technologies are instrumental in exploring the transcriptional, proteomic, and metabolic landscapes of OA-affected tissues. Bulk RNA sequencing analyzes the collective gene expression of mixed cell populations from tissues. This method provides an overall picture of the transcriptional activity in a sample, such as synovial fluid, cartilage, or bone from OA patients.^50^ Single-cell RNA sequencing (scRNA-seq) is a high-throughput technology offers a high-resolution view of cellular diversity by analyzing the gene expression of individual cells. This technique is crucial for identifying specific cell types and states within heterogeneous tissues, such as inflamed synovial tissue or degenerating cartilage in OA.^51^

#### Omics analysis

Gene expression data from key affected sites (synovium, cartilage, subchondral bone) reveal differential gene expression patterns. These patterns help to understand the transcriptional changes associated with disease progression. Single-cell RNA sequencing, in particular, allows for the high-resolution analysis of these changes at the cellular level, helping to identify cellular subtypes and their roles in OA.^52^ Protein profiles from tissues and fluids, such as synovial fluid, identify critical proteins involved in the disease process. This approach provides insights into the proteomic landscape of OA, highlighting proteins that contribute to cartilage degradation and joint inflammation.^53^ Metabolic profiling of biological fluids like blood and synovial fluid helps to identify shifts in metabolic pathways that are symptomatic of OA, offering potential biomarkers for the disease.^54^ Integrated omics analysis helps map out the complex, interrelated signaling pathways and identify biomarkers for diagnosis and prognosis. This comprehensive approach is essential for developing targeted therapies and improving our understanding of OA pathogenesis.

#### Limitations of traditional methods

Traditional statistical models often assume linear relationships between variables, which is overly simplistic for omics data involving complex, nonlinear interactions. These models may not fully capture the underlying biological processes, leading to potentially misleading conclusions.^55^ Omics datasets often contain a vast number of features relative to sample size, leading to challenges such as overfitting and difficulties in model selection and inference.^56^ Integrating data from different omics levels (genomics, proteomics, metabolomics) is often challenging with traditional methods, which may not effectively analyze the interactions between these layers to provide a comprehensive understanding of OA.^57^ Traditional statistical methods might not scale efficiently with the increasing size and complexity of omics data, leading to increased computational demands and longer processing times.^58^ Traditional models often fail to account for temporal and spatial variations in disease progression, which are vital for understanding the dynamics of OA.^59^

## Data-driven applications of Artificial Intelligence in osteoarthritis

### Applications of AI in clinical data

Clinical data in OA research is characterized by large volumes, a wide variety of data types, and features that often remain at a superficial level. AI has the potential to uncover complex, underlying relationships within these features.^60^ For instance, EHR, basic physiological information, and demographic data offer valuable insights into a patient’s historical health profile. However, traditional methods often struggle to reveal the intricate interconnections among these features.^61^

**Fig. 1:**
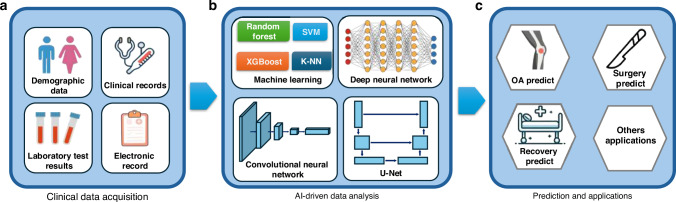
Workflow for clinical data analysis and AI-driven applications in OA: the process begins with data acquisition from various clinical sources, including basic demographic data, clinical visit records, laboratory test results, and electronic medical records (**a**). These data are analyzed using machine learning (e.g., Random Forest, SVM, XGBoost, K-NN) and deep learning methods (e.g., deep neural networks, convolutional neural networks, U-Net) to identify latent pathological features and risk factors associated with OA (**b**). The resulting insights are applied to a range of clinical tasks, such as OA risk prediction, surgery prediction, recovery prediction, and other applications, facilitating early screening, personalized treatment strategies, and improved patient outcomes (**c**)

The integration of AI offers a promising solution, leveraging machine learning and deep learning methods to identify latent pathological features within large-scale EHR data that may correlate with OA risk, thereby enabling the development of personalized risk assessment models.^62^ As shown in Fig.1, AI-driven analysis of these data sources could facilitate early screening and prediction of OA without additional imaging or histological examinations. Instead, standard physiological screenings, past medical records, and demographic information alone could suffice.^63,64^ This approach not only reduces the diagnostic burden on patients but also increases the efficiency of early OA screening, paving the way for cost-effective management strategies in OA care.^65^ Ningrum et al. built a deep-learning model to predict the risk of developing knee arthritis within the next year. Important features for predicting knee osteoarthritis (KOA) were screened based on their influence on prediction accuracy. These included diseases associated with the eye and adnexa, acute respiratory infections, conditions of the esophagus, stomach, and duodenum, musculoskeletal and connective tissues, chronic comorbidities (e.g., metabolic disorders, immunity-related conditions, circulatory system issues, and hypertension-associated disorders), and medications such as antacids, cough suppressants, and expectorants.^66^

Yau et al. focused on predicting OA development risk through machine learning techniques such as support vector machines (SVM) and decision trees applied to EHRs.^67^ Logistic regression was employed by Jason et al. to construct a prediction model estimating the 5-year risk of developing OA using the entire cohort.^68^ Chen et al. utilized Random Forest, XGBoost (XGB), SVM and K-nearest neighbors (K-NN) to build a prediction model from primary care electronic medical records, aiming to estimate the 5-year risk of OA development.^69^ Random forest, XGBoost, and K-NN are commonly used machine learning algorithms. They predict outcomes by constructing multiple decision trees (random forest), optimize model performance through gradient boosting (XGBoost), and classify or regress based on the distances between data points (K-NN). Specifically, Extreme Gradient Boosting (XGBoost) is a machine learning algorithm based on gradient-boosted decision trees. By integrating multiple weak classifiers, improves prediction accuracy and is suitable for high-dimensional data classification and regression tasks.

Deep neural networks (DNN) combined with scaled principal component analysis were used by Lim^70^ to automatically generate features from data and identify risk factors for OA prevalence. Similarly, Christodoulou et al. leveraged DNN within EHRs to effectively identify OA cases, thereby enhancing capabilities for early diagnosis.^71^

Nielsen et al. utilized XGBoost to determine that higher age, BMI, and prescription of non-steroidal anti-inflammatory drugs were the most significant predictors of increased OA risk ahead of diagnosis.^72^ Another study by Zhou et al. reduced predictors and associated variables using a Random Forest method, generating decision rules from a decision tree model. Out of a total of 43 100 variables, 900 predictors were reduced to 37 groups of related clinical codes, which were then used to develop a decision tree model.^73^

In addition to early screening, clinical data can also be utilized as input for predictive models to assess OA disease progression, evaluate the necessity for surgical intervention, and forecast postoperative recovery outcomes. Castagno et al. developed an automated machine learning tool to predict the rapid progression of knee OA over a 2-year period.^74^ Building upon previous work, Hancox et al. used temporal graph convolutional neural network models to construct temporal graphs from primary care medical event codes.^75^ These codes, sourced from ResearchOne EHRs of 40–75-year-old patients, were used to predict the risk of hip replacement.

Crawford et al. designed a machine learning algorithm to identify potential surgical candidates for joint arthroplasty in OA patients without requiring an in-person evaluation or physical examination.^76^ Park further refined this approach by incorporating 104-episode care characteristics and prospective patient-reported outcome measures into logistic regression models with least absolute shrinkage, selection operator (Lasso) penalty, and random forest algorithms for prediction performance evaluation and comparison.^77^

Kunze et al. created a clinical decision-making tool leveraging partially modifiable risk factors to predict clinically significant outcomes following total hip arthroplasty (THA).^78^ Harris et al. developed models that estimate patient-specific improvements in major outcomes 1 year after total knee arthroplasty. Integrating these models into clinical decision support systems enhances “informed consent processes, shared decision-making, and patient education, ultimately improving” patient selection and satisfaction.^79^

By analyzing patient histories, physiological metrics, and demographic information, these models offer customized insights into OA progression and treatment efficacy, assisting clinicians in crafting more informed treatment plans and post-surgical care strategies. This expanded application of clinical data underscores its potential to inform comprehensive OA management, spanning preventive care, surgical decision-making, and recovery planning.

Moreover, AI’s utilization of clinical data goes beyond basic diagnostics and treatment predictions in OA. It has proven effective in distinguishing between rheumatoid arthritis (RA) and OA—a critical step for treatment-specific strategies. AI also predicts the likelihood of OA patients developing other comorbidities, thereby enhancing overall patient management.^80,81^ Additionally, AI algorithms assess pain levels and identify patients who may not adequately respond to standard pain medications. This capability supports the development of more personalized and effective pain management strategies.^82^

By harnessing deep insights from clinical data, AI enables a comprehensive approach to patient care, improving outcomes across multiple health dimensions and ensuring tailored, patient-specific interventions.

### Applications of AI in image data

Artificial intelligence (AI) has emerged as a transformative tool in the analysis and application of imaging data for OA, significantly enhancing diagnostic accuracy and operational efficiency. Traditional imaging workflows often depend on manual image processing and expert interpretation, which introduces potential delays, subjective variability, and challenges in identifying subtle, early-stage changes. In contrast, AI-driven methods streamline image acquisition, processing, and interpretation, providing predictive capabilities while improving both speed and diagnostic consistency.

**Fig. 2:**
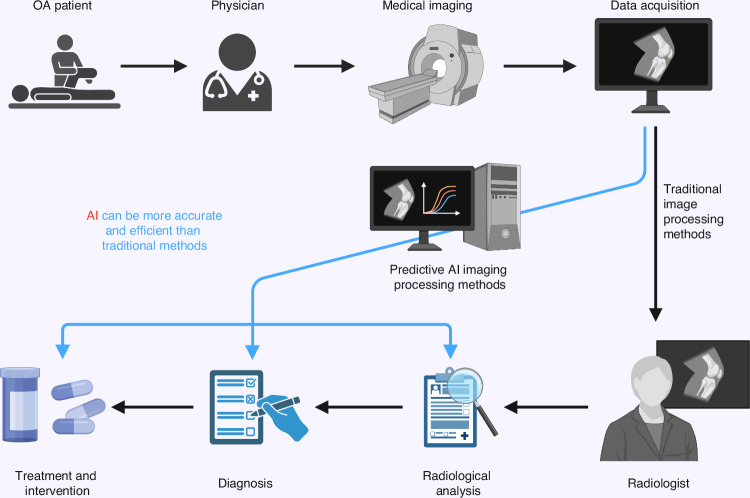
Workflow of AI-enhanced diagnosis and treatment of osteoarthritis: from image acquisition to rapid clinical decision support. AI first performs preprocessing of the raw imaging data. The algorithm automatically extracts key information from the joint structures, such as morphological changes in cartilage and bone, and identifies potential lesion areas. Deep learning models are used to detect abnormal structures within the joint at the early stages of OA. Finally, AI technology integrates clinical and omics data to provide a more comprehensive assessment of OA progression. By enhancing the disease diagnosis and treatment workflow with AI, the sensitivity of early OA screening can be significantly improved, and personalized treatment plans can be developed

As illustrated in Fig. 2, AI integration redefines the OA imaging workflow. From the moment a patient undergoes imaging—such as MRI or X-ray acquisition—AI algorithms can automatically handle tasks such as image preprocessing, feature extraction, and pattern recognition. Instead of relying solely on a radiologist’s interpretation, AI imaging methods rapidly identify structural abnormalities, detect small-scale cartilage or bone changes, and highlight early signs of joint degeneration. Moreover, these methods integrate with clinical and omics data, enabling a holistic view of OA progression.

By leveraging deep learning architectures, CNNs, and other machine learning frameworks, AI-driven image analysis enhances diagnostic accuracy, improves reproducibility, and minimizes interobserver variability. The following sections detail the specific applications of AI across imaging modalities, highlighting advances in X-ray, MRI, CT and US analyses, as well as the integration of multimodal data and end-to-end AI-driven solutions.

#### Automated X-ray image analysis

AI algorithms, particularly deep learning models such as CNNs, can automatically identify and quantify key features in X-ray images, including osteophytes, joint space narrowing, and bone remodeling. By training these models on extensive datasets, they excel in feature extraction and classification, thereby accelerating diagnostics, enhancing detection precision, and reducing human error.^83^

#### Enhanced MRI image processing

AI technologies are extensively applied in MRI image analysis, addressing processes like image segmentation, feature extraction, and pattern recognition. Utilizing deep learning and CNNs, AI can detect subtle OA changes such as cartilage thinning and bone marrow lesions at earlier stages. Machine learning models trained on large-scale datasets automate the recognition of abnormal patterns, offering robust support for clinical diagnostics and enabling earlier interventions.^84^

#### CT image reconstruction and bone density analysis

AI plays a crucial role in three-dimensional reconstruction and bone density analysis of CT images. Using deep learning algorithms, AI automates the reconstruction of 3D bone structures, accurately measures bone density, and identifies even minor osseous changes. This capability is instrumental in assessing subchondral bone integrity, evaluating OA severity, and supporting bone mineral density quantification.^85^

#### Ultrasound image analysis

AI integration into real-time US imaging enhances the analysis of soft tissue conditions, such as the extent of synovitis and joint effusions. AI-assisted technologies, including real-time object detection and localization algorithms, improve the precision and safety of surgical procedures like intra-articular injections. These technologies employ machine learning models for real-time data processing, ensuring efficient, accurate, and safer clinical operations.^36,86^

#### Enhanced advanced imaging analysis

Although traditional high-resolution imaging techniques (such as second harmonic generation microscopy and confocal laser scanning arthroscopy) can visualize the multilayer ultrastructure of cartilage.^87^ Their analysis is reliant on manual annotation and is time-consuming.^88^ AI technology can optimize this process through auto-matric segmentation and quantification. U-Net-based models can automatically segment the superficial, middle, and deep layers of cartilage, allowing for precise measurement of layer thickness and collagen arrangement. For micro-damage detection, CNNs can identify early microcracks and proteoglycan loss from electron microscopy images. Furthermore, by combining AI with finite element analysis, cartilage stress distribution under mechanical loading can be predicted, guiding dynamic modeling and personalized interventions. These advancements not only enhance diagnostic accuracy but also have the potential for translation into intraoperative navigation tools (such as AI-guided real-time cartilage repair surgery) and drug efficacy assessment platforms (such as AI-based quantification of cartilage regeneration in organoid models).

Imaging data are essential for clinicians diagnosing OA, providing a deeper insight into the underlying causes and severity of the condition compared to standard clinical data. Unlike routine physiological and demographic information, imaging data can capture structural and morphological changes in joint tissues that are critical for assessing OA. Recently, numerous AI research initiatives have focused on automating the end-to-end analysis of imaging data to enhance diagnostic precision and reliability in OA assessments. These AI-driven approaches can assist clinicians by analysing imaging data to detect early signs of OA, such as joint degradation, with high accuracy, contributing to more informed treatment decisions and potentially even replacing manual evaluations for certain diagnostic tasks.^89^ This progress highlights AI’s capacity not only to support but also to transform OA diagnosis, promoting timely and accurate intervention strategies and broadening the scope of predictive analysis in OA care.^90^

**Fig. 3:**
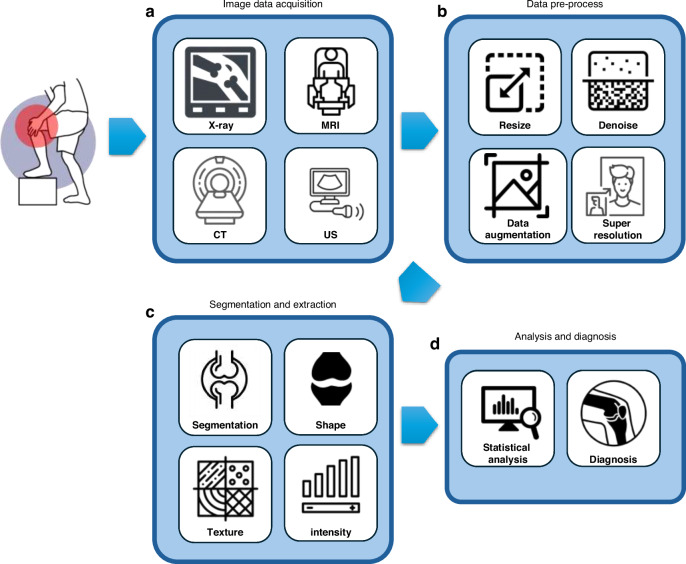
Traditional workflow for OA image data analysis and diagnosis. **a** Acquire imaging data (e.g., X-ray, MRI, CT, ultrasound) from clinical sources. **b** Preprocess images (resizing, denoising, data augmentation) and perform segmentation. **c** Extract relevant features (e.g., texture, shape). **d** Statistical analysis and integrate results into clinical decision support

As illustrated in Fig. 3, the analysis and diagnosis of OA from imaging data typically follow a generally consistent and well-established workflow, encompassing preprocessing, segmentation of regions of interest, extraction of texture features, and ultimately, OA diagnosis. Traditional methods have long provided clinicians with reliable protocols for each step of imaging analysis. However, AI now enhances this process by contributing to each phase, offering new capabilities in data handling and analysis. Table 1 summarizes AI applications at each stage, from initial data preprocessing to final diagnostic assessment. Some AI-driven approaches have even streamlined this process into an end-to-end solution, bypassing the need for manual intervention and directly transforming raw imaging data into diagnostic insights. Such advancements not only reduce the complexity of the workflow but also promise to enhance diagnostic accuracy and efficiency, offering significant support to clinicians.

**Table 1:** Comparison of AI applications in OA field using image data

Preprocess	Segment	Feature extraction	Analysis	Description	Modality	Model	Authors
√	-	-	-	Image reconstruction	MRI	Multiscale ResNet	Hu et al.^107^
√	-	-	-	Image optimization	US	Back propagation (BP) neural network algorithm	Zhao et al.^108^
-	√	√	√	Semi-automatically extracted radiomic features from tibial subchondral bone to diagnose knee OA	MRI	Multi-atlas and appearance models, Workflow for Optimal Radiomics Classification, Elastic Net	Hirvasniemi et al.^109^
-	√	-	-	Fully automatic method for segmentation	MRI	U-Net	Panfilov et al.^110^
-	√	-	√	Knee OA detecting	X-ray	U-Net, ResNet-50	Li et al.^111^
-	√	-	√	Locating and grading knee OA	X-ray	Faster RCNN and ResNet-50, AlexNet	Abdullah et al.^112^
-	√	-	-	the concurrent segmentation of the distal femur and the proximal tibia	CT	2D, 3D U-Net	Marzorati et al.^113^
-	√	-	-	the segmentation of lateral joint space and medial joint space (LJS and MJS) of knee joints	CT	DeepRegY based on RegNet and DeepLabV3P, a new attention mechanism called “Feature-Location” module	Shen et al.^114^
-	√	-	-	automatically segment 3D ultrasound images of the synovial tissue in osteoarthritis of the first carpometacarpal (CMC1 OA)	US	2D U-Net	Sasaki et al.^115^
-	√	-	√	the segmentation of disease regions and the automated identification of the typical knee joint diseases	US	Deeplabv3, optimized-classification-based Resnet	Long et al.^116^
-	-	√	√	Distinguishing OA and non-OA from X-ray images	X-ray	“Discriminative shape-texture convolutional neural network (DST-CNN)”	Nasser et al.^117^
-	-	√	√	Diagnose OA in knee X-ray images at an early stage	X-ray	2D-CNN, random forest and k-NN	Rehman et al.^118^
-	-	-	√	Enhancing the Accuracy of MRI Imaging for OA Diagnosis	MRI	3D CNN	Guida et al.^119^
-	-	-	√	Stratifying knees into MRI-based morphological phenotypes for helping predicting future OA incidence	MRI	MRNet	Namiri et al.^120^
-	-	-	√	Predicting OA progression	MRI	DeepKOA, base on DenseNet169	Hu et al.^121^
-	-	-	√	Identify significant structural factors associated with pain severity in KOA patients	MRI	CNN, class activation mapping (CAM)	Zhao et al.^122^
-	-	-	√	Explore the diagnostic value in hip osteoarthritis (HOA)	X-ray	Deep CNN, vgg-16	Xue et al.^123^
-	-	-	√	Accelerate the classification of KOA severity	X-ray	Deep convolutional neural networks (DCNN)	Tri Wahyuningrum et al.^124^
-	-	-	√	Evaluate how well an AI can classify the severity of knee OA	X-ray	ResNet	Olsson et al.^125^
-	-	-	√	OA prediction at early stage	X-ray	GMDH-type neural networks	Jakaite et al.^126^
-	-	-	√	Automated grading of knee OA	X-ray	CNN with attention module	Feng et al.^127^
-	-	-	√	Identify and classify KOA in an automated, faster, and accurate manner	X-ray	VGG16, VGG19, ResNet101, MobileNetV2, InceptionResNetV2, and DenseNet121	Mohammed et al.^128^
-	-	-	√	The early detection of knee OA	X-ray	Sequential CNNs, VGG-16 and ResNet-50	Alshamrani et al.^129^
-	-	-	√	Classify KOA into five categories	X-ray	CNN	Sajaan et al.^130^
-	-	-	√	Predict knee OA grades with fusion features	X-ray	VGG-19	Khalid et al.^91^

In addition to commonly used imaging modalities, we observe applications involving cellular and tissue-level image data for OA analysis. For instance, Mehta et al. utilized H&E-stained synovial tissue samples from total knee replacement (TKR) explants as input, employing a random forest model to assess how OA and RA uniquely affect cellular characteristics, ultimately distinguishing between OA and RA patients based on these differences.^91^

Furthermore, multimodal input—where both image and non-image data are combined—has become increasingly prevalent. An example of this is the work of Karim et al., who integrated MRI and X-ray images as inputs for two separate deep-learning models. Through ensemble learning, their approach demonstrated enhanced diagnostic accuracy for OA by leveraging the complementary information from multiple imaging sources.^92^ And for the first time, Guida et al. propose a fusion model that combines three different modalities (X-ray, MRI, and the patient’s clinical information) into one network to improve the accuracy over the models being used individually.^93^ This evolution not only alleviates clinicians’ workload but also holds the potential to improve diagnostic accuracy and accelerate patient care.

### Applications of AI in omic data

**Fig. 4:**
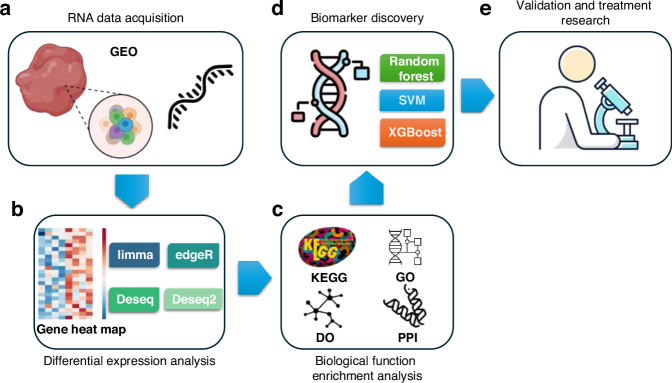
Workflow for OA transcriptomic biomarker discovery. **a** Obtain scRNA-seq and bulk RNA-seq datasets from databases like GEO. **b** Identify differentially expressed genes using analytical tools (e.g., limma, edgeR, DESeq2). **c** Perform functional and pathway enrichment analysis (e.g., KEGG, GO, DO, PPI). **d** Identify genes associated with OA diagnosis by machine learning models (e.g., Random Forest, SVM, XGBoost). **e** Validate biomarkers in other datasets and investigate potential treatments

As a more granular, cell-level data source, omics data provides insights beyond imaging data, offering invaluable information for understanding the underlying mechanisms of OA. Omics analyses are particularly useful for identifying the molecular basis of OA, revealing its complex pathophysiology at a cellular.^94,95^ A common approach of transcriptomics in the field of OA is the identification of differentially expressed genes (DEGs) in OA patients, followed by functional enrichment analysis using methods such as Kyoto encyclopedia of genes and genomes, gene ontology, disease ontology, and protein-protein interaction to identify OA-related signaling pathways and biological processes. Huang et al.^96^ innovatively applied gene deconvolutional methods and the machine learning tool CIBERSORTx to effectively reveal the distribution of different cell types in the synovium. Compared to traditional methods, this approach enhanced the accuracy of characterizing osteoarthritic synovial tissue, offering new insights into the pathological changes of OA. Additionally, machine learning methods are employed to select biomarkers that can be used for OA classification,^97,98^ as illustrated in Fig. 4. Further downstream analyses may involve validation experiments and exploration of potential therapeutic drugs. Some studies, in contrast to the approaches mentioned earlier, specifically focus on posignaling pathways and functions. These studies perform functional enrichment analysis to identify genes associated with specific functions and then examine the overlap between these genes and the DEGs in OA patients. Machine learning methods are subsequently used to discover corresponding biomarkers. Zhao et al.^99^ utilized machine learning and transcriptomics to identify four key genes (CRTAC1, DIO2, ANGPTL2, MAGED1) closely associated with OA cartilage inflammation and immune response. The upregulation of these genes is correlated with immune cell infiltration and markers of cartilage degeneration and bone mineralization, providing potential molecular markers for the early diagnosis and targeted treatment of OA. Table 2 provides examples of such approaches. Compared to traditional clinical biomarkers (such as inflammatory factors IL-6, TNF-*α*, and cartilage degradation markers COMP, CTX-II), AI-driven transcriptomic biomarkers (such as RIPK3 and PDK1) have the following advantages: (1) Higher specificity: AI enables the analysis of gene expression profiles in specific cell subpopulations (e.g., chondrocytes, synovial macrophages) with single-cell resolution; (2) Dynamic monitoring potential: AI models can integrate time-series omics data to capture molecular changes during disease progression; (3) Multifactorial associations: AI can identify regulatory networks directly related to OA pathology (such as inflammation and metabolic disturbances), whereas traditional biomarkers are often limited to single-phenotype indicators. For example, the chondrocyte necrosis-associated gene RIPK3 (Table 2), discovered by AI, is directly linked to cartilage matrix degradation, whereas the traditional biomarker CTX-II only reflects the overall collagen metabolism level. Overall, AI-driven biomarkers focus more on cell-specific mechanisms (such as chondrocyte necrosis and macrophage polarization), while existing biomarkers are typically systemic indicators or tissue-specific metabolic products. Additionally, some studies have utilized bulk RNA-seq data, which is used to investigate the heterogeneity of synovial cells.^96^ Wu et al.^100^ integrated multi-omics data with machine learning methods to identify critical mitochondrial function-related genes and explored their significant role in the progression of OA, suggesting that these genes may serve as potential targets for personalized treatment of OA. By leveraging the precision of transcriptomic data, AI offers a pathway to deeper insights and innovative treatment strategies for OA.

**Table 2:** AI-driven biomarker (signature gene) discovery in various transcriptomic directions

Years	Target	Signature genes	Existing clinical biomarkers	Methodology	Authors
2024	Mitochondria-related	GRPEL and MTFP1	Bone metabolism biomarkers: COMP (cartilage oligomeric matrix protein), CTX-II (C-terminal telopeptide of type II collagen)	LASSO, SVM-RFE	Wang et al.^131^
2024	Tryptophan metabolism related	TDO2, AOX1 and SLC3A2	Inflammatory biomarkers: IL-6, CRP (C-reactive protein)	NB, SVM-RFE	Yang et al.^132^
2024	Chondrocyte necroptosis	RIPK3, CYBB, HSP90AB1 and TRAF5	Cartilage degradation biomarkers: GAGs (Glycosaminoglycans), CSase (Chondroitinase)	LASSO	Deng et al.^133^
2024	Hypoxia-related	ADM, DDIT3 and MAFF	Bone formation biomarkers: BAP (Bone-specific alkaline phosphatase), OCN (osteocalcin)	LASSO, RF	Liu et al.^134^
2024	Macrophage polarization-related genes	CSF1R, CX3CR1, CEBPB and TLR7	Inflammation and immune biomarkers: TNF-*α*, IL-1*β*	LASSO, SVM	Hu et al.^135^
2024	OA immune microenvironment	FCER1G, HLA-DMB, and HHLA-DPA1	Immune modulatory biomarkers: IL-10, IFN-*γ*	SVM-RFE, RF	Pang et al.^136^
2024	Osteoblastic autophagy-related	DDIT3, JUN and VEGFA	Bone resorption biomarkers: PYD (pyridinoline), HYP (hydroxyproline)	Boruta, ETC, SVM-RFE, RF	Cai et al.^137^
2024	Differential anoikis-related genes	CDH2, SHCBP1, SCG2, C10orf10, PFKFB3	Systemic inflammation biomarkers: ESR (erythrocyte sedimentation rate), CRP	XGB, SVM-RFE, RF, GLM	Zhang et _al_. ^138^
2024	Endoplasmic reticulum related genes	HSPA5, UBL4A, ATF4, PPP1R15A	Cellular stress biomarkers: HSP70 (heat shock protein 70), CHOP (C/EBP homologous protein)	LASSO, RF	Liu et al.^139^
2023	Aging-related biomarkers	MCL1, SIK1, JUND, NFKBIA and JUN	Senescence-associated secretory phenotype (SASP): IL-6, IL-1*β*	LASSO, SVM-RFE, RF	Zhou et al.^140^
2023	Immune-related	FZD7, IRAK3, KDELR3, PHC2, RHOB, RNF170, SOX13, and ZKSCAN4	Combined detection biomarkers: IL-6 + PCT (Procalcitonin)	LASSO, RF	Li et al.^141^
2023	Chondrocyte autophagy and apoptosis	PDK1	Cartilage metabolism biomarkers: COL2A1 (collagen type II alpha1 chain), MMP-13 (matrix metalloproteinase 13)	LASSO, SVM-RFE	Meng et al.^142^
2022	Macrophage- associated genes	IL1B, C5AR1, FCGR2B, IL10, IL6 and TYROBP	Macrophage polarization biomarkers: CD68 (macrophage marker), CD163 (M2 macrophage marker)	LASSO, RF	Liu et al.^143^
2022	Pathological immune cell infiltration	GUCA1A and NELL1	Synovial inflammation biomarkers: VEGF (vascular endothelial growth factor), MMP-3 (matrix metalloproteinase 3)	LASSO, SVM-RFE, RF	Liu et al.^144^

*LASSO* least absolute shrinkage and selection operator, *SVM-RFE* support vector machine-recursive feature elimination, *RF* random forest, *NB* naive bayes, *Boruta* boruta algorithm, *ETC* extra trees classifier, *XGB* extreme gradient boosting, *GLM* generalized linear model, *SVM* support vector machine

Beyond the aforementioned transcriptomics, AI has also contributed to advancing the understanding of OA through applications in other omics fields. In proteomics, Tardif et al. employed machine learning to validate differential proteins in OA patients, offering insights into protein biomarkers that could enhance OA diagnostics and therapeutic strategies.^101^ In the field of epigenomics, Dunn et al. developed epigenetic biomarker models ”based on” peripheral blood samples to predict knee OA progression, emphasizing the impact of epigenetic modifications on early prognostic assessments.^102^ In metabolomics, Werdyani et al. used logistic regression models to identify key metabolites linked to OA endotypes, advancing the understanding of OA’s heterogeneity.^103^ Additionally, Bocsa et al. applied high-performance liquid chromatography coupled with untargeted mass spectrometry (LC-MS) to profile the metabolomic differences between early and late-stage OA, revealing metabolic variations specific to disease stages that could guide targeted interventions.^104^

## Discussion and conclusion

OA research has historically faced several significant challenges in diagnosis, disease progression prediction, and therapeutic intervention. Traditional clinical, imaging, and omics data face various limitations in OA research, which hinder early detection, disease monitoring, and personalized treatment. Clinical data often lack sufficient pathological depth, making it difficult to uncover early OA risks and complex interrelationships.

Conventional imaging methods, such as X-ray and MRI, are unable to detect early soft tissue changes or capture subtle joint progression, and they heavily rely on expert interpretation, leading to potential diagnostic variability. Omics data, while offering valuable insights into the molecular mechanisms of OA, are often complex and high-dimensional, requiring sophisticated analysis techniques that are difficult to integrate directly into clinical applications. These methods also rely heavily on clinician expertize, leading to interobserver variability and inconsistencies in diagnosis.

Furthermore, existing treatment options primarily focus on symptom management rather than addressing the underlying disease processes, underscoring the need for more advanced diagnostic tools as well as personalized therapies. These limitations highlight the urgent need for integrating multiple data sources and applying AI to improve diagnostic accuracy and enhance treatment strategies.

AI has emerged as a powerful solution to many of these challenges, providing innovative methods to enhance both the diagnosis and management of OA. One of the most significant advantages of AI in OA research is its ability to analyze large, complex datasets—such as medical imaging, genetic data, and clinical records—more efficiently and accurately than traditional methods.

AI-powered deep learning models, particularly CNNs, have demonstrated the ability to identify subtle structural changes in joints, detecting early signs of OA that are frequently overlooked by human observers. Additionally, AI has shown promise in automating the grading of OA severity, reducing interobserver variability, and providing more consistent, objective assessments.

Machine learning algorithms also enable the integration of diverse data types, such as clinical, genetic, and biomechanical information, to predict OA progression and identify high-risk patients, thereby facilitating earlier detection and more personalized interventions.

Current OA therapeutic targets (such as BMP-7 and FGF-18) primarily focus on cartilage anabolic metabolism or inflammation inhibition,^105^ whereas AI-driven targets (such as PDK1 and RIPK3) reveal new mechanisms. For example, in the regulation of cell death, RIPK3-mediated chondrocyte necrosis provides a novel direction for targeting programmed necrosis. In terms of metabolic reprogramming, PDK1 influences chondrocyte survival through the regulation of glycolysis, distinguishing it from traditional growth factor targets. AI models can also predict the synergistic effects of multi-target combinations (e.g., IL-1 inhibitors + RIPK3 antagonists), accelerating the development of combination therapies. Additionally, AI can optimize the molecular design of existing targets (e.g., FGF-18 analogs) through virtual drug screening, improving efficacy and safety. AI has been instrumental in advancing drug discovery for OA by identifying novel therapeutic targets and repurposing existing drugs for new indications. This opens up new avenues for more effective treatments, particularly for earlier stages of OA where current options are limited. Moreover, in the field of omics, AI has contributed to the identification of biomarkers for OA diagnosis, prognosis, and treatment response. For example, AI-based analyses of bulk and single-cell RNA sequencing data have revealed differential gene expression patterns and identified key molecular pathways involved in OA progression, providing valuable insights into the disease’s underlying biology.

The heterogeneity of OA arises from the interplay of multiple cell types (chondrocytes, synovial fibroblasts, immune cells) and pathological mechanisms (inflammation, metabolic imbalance, mechanical injury).^106^ AI can address this complexity through multimodal data integration strategies, utilizing combined X-ray, MRI, and clinical data to identify correlations between bone marrow edema and synovial metabolic genes. In terms of cell type-specific biomarkers, single-cell transcriptomics combined with AI can localize the dynamic contributions of macrophage subgroups (M1/M2) in OA progression. Additionally, in imaging-genomics combined biomarkers, for example, the deep learning model DeepKOA can associate MRI cartilage texture features with plasma metabolites to predict subtype-specific progression risks. These integrated approaches lay the foundation for developing precise classification systems (e.g., inflammatory vs. mechanical OA).

However, the rapid advancement of AI in OA research also brings several challenges that need to be addressed. One of the major concerns is the lack of interpretability and transparency in many AI models. While these models may achieve high accuracy, their opaque nature makes it difficult to understand the rationale behind their predictions, which limits their clinical applicability. In medical contexts, where decisions directly affect patient health, it is crucial for AI models to be interpretable and explainable. Additionally, the widespread use of AI in healthcare raises concerns about data privacy and security. Patient data, especially genetic and clinical information, is highly sensitive, and the risk of data breaches or misuse must be carefully managed.

Furthermore, AI’s reliance on large, high-quality datasets can introduce biases if the data used for training models are not representative of diverse patient populations. This could lead to disparities in the accuracy and fairness of AI-based tools, particularly for underrepresented groups. Ensuring that AI models are trained on diverse datasets and that their performance is evaluated across different demographic groups is essential for their equitable application in clinical settings.

Looking to the future, AI has the potential to revolutionize OA research and clinical practice further. As AI models become more sophisticated and interpretable, they could provide not only early and accurate diagnoses but also real-time monitoring of disease progression, allowing for continuous, personalized management of OA. The integration of AI with emerging technologies can enable patient-specific dynamic therapeutic strategies. The combination of AI and wearable sensors, such as inertial measurement units and smart joint patches, will facilitate real-time monitoring of OA progression. For instance, by analyzing gait data and joint load distribution, AI models can dynamically assess cartilage degeneration risks and provide a foundation for personalized rehabilitation plans. In the domain of AI-driven organ-on-a-chip and drug screening, combining AI with bone/cartilage organ technology enables the construction of high-throughput drug screening platforms to simulate the OA microenvironment and predict treatment responses. Furthermore, privacy concerns associated with cross-institutional data sharing can be addressed through federated learning frameworks. For example, distributed training models allow for the integration of multi-center imaging and omics data while protecting patient data privacy, thereby enhancing the model’s generalization capabilities. In addition, AI could facilitate the discovery of new biomarkers and therapeutic targets, leading to the development of disease-modifying treatments that address the root causes of OA rather than merely alleviating symptoms.

The combination of AI and omics technologies holds particular promise for uncovering the complex molecular mechanisms of OA and identifying novel biomarkers for diagnosis and treatment. AI could facilitate the integration of multi-omics data (genomics, proteomics, metabolomics) to provide a more comprehensive understanding of OA pathogenesis and guide the development of personalized treatment plans. Furthermore, AI-driven models could aid in the development of precision medicine approaches, tailoring treatments based on an individual’s unique molecular profile, genetic predisposition, and disease progression.

In conclusion, AI has the potential to overcome many of the longstanding challenges in OA research and clinical practice, providing more accurate, timely, and personalized diagnostic and treatment solutions. However, to fully realize the benefits of AI, it will be necessary to address challenges related to model transparency, data privacy, and bias. As AI continues to evolve, it is likely to play an increasingly central role in shaping the future of OA diagnosis, treatment, and management, paving the way for more effective and individualized patient care.
